# GLI 2012 equations define few spirometric anomalies in the general population: the PneumoLaus study

**DOI:** 10.1186/s12931-018-0955-0

**Published:** 2018-12-13

**Authors:** Alexandra Lenoir, Jean-William Fitting, Pedro-Manuel Marques-Vidal, Peter Vollenweider, Laurent P. Nicod

**Affiliations:** 1 Department of Medicine, Respiratory Medicine, Lausanne University Hospital (CHUV), Rue du Bugnon 46, 1011 Lausanne, Switzerland; 2 Department of Medicine, Internal Medicine, Lausanne University Hospital (CHUV), Lausanne, Switzerland

**Keywords:** Spirometry, Population study, Airway obstruction, Lung restriction, GLI 2012, Smoking

## Abstract

**Background:**

Reduced lung function predicts increased mortality, but its prevalence may vary depending on definition considered, use of bronchodilation and applied reference values. We aimed to assess lung function abnormalities in Lausanne, Switzerland, and their association with clinical history.

**Methods:**

In a general population sample, spirometry was performed and bronchodilation applied if the ratio forced expiratory volume in 1 s (FEV1) / forced vital capacity (FVC) or the FVC was below the lower limit of normal (LLN) according to Global Lung Function Initiative 2012 references. Results for FEV1/FVC according to the LLN were compared to the 0.7 fixed ratio. Respiratory risk factors, symptoms and self-reported respiratory diagnoses were recorded through a questionnaire.

**Results:**

Out of the 3342 included subjects, 3.8% had chronic obstruction and 2.5% reversible obstruction when using the LLN; possible lung restriction alone was present in 1.8%, and associated with chronic obstruction in 0.4%. Ever smokers had a higher prevalence of abnormal spirometry, chronic obstruction and reversible obstruction; there was no difference with regard to possible restriction. Overall, chronic airway obstruction was found in 8.9% of current smokers, 4.6% of former smokers and 1.5% of never smokers. Only one third of participants with chronic obstruction were aware of a respiratory disease.

**Conclusion:**

Prevalence of abnormal lung function in the population of Lausanne is low. This may be due to a low rate of ever-smokers, the application of a full bronchodilation dose, but also to inherent characteristics of this population.

## Introduction

A reduced lung function, as expressed by the forced expiratory volume in one second (FEV1) and the forced vital capacity (FVC), has been shown to be a marker of premature death, mainly through cardiopulmonary complications [1, 2]. The definition of abnormal spirometry results has however been subject to debate, especially the use of a fixed FEV1/FVC ratio versus a lower limit of normal (LLN) to diagnose airway obstruction [3, 4]. Furthermore, the use of bronchodilation is not uniform across studies. Finally, different reference values have been used, the European Community of Coal and Steel (ECCS) reference equations. [5], the American National Health and Nutrition Examination Survey (NHANES) III reference values [6], and eventually the more recent and comprehensive Global Lung Function Initiative (GLI) 2012 reference equations. [7].

Given the variety of methods and reference equations cited above, the currently available data on prevalence of abnormal lung function in general populations are difficult to compare.

The primary objective of the PneumoLaus study was to assess the prevalence of lung function abnormalities in the population of the city of Lausanne, Switzerland, according to GLI 2012 reference values [7], using bronchodilation in case of airway obstruction. The secondary objectives of our study were to assess in the same population if a higher prevalence of lung function abnormalities was associated with risk factors for respiratory diseases, respiratory symptoms or self-reported history of respiratory diagnoses.

## Methods

### Setting and selection of participants

The PneumoLaus study is a part of the CoLaus/PsyCoLaus study, a population-based, prospective study investigating the prevalence and determinants of cardiovascular disease in the city of Lausanne, Switzerland. The sampling procedure of the CoLaus/PsyCoLaus study has been described previously [8]. In brief, all subjects aged 35–75 years living in the city of Lausanne were eligible, and a simple, non-stratified random sample of 6733 participants was obtained using the population register of the city and examined between June 2003 and May 2006. The first follow-up was conducted between April 2009 and September 2012, and the second follow-up was conducted between May 2014 and April 2017.

Starting in June 2014, all participants were invited to take part in the PneumoLaus study. Examinations were conducted between June 2014 and August 2017. All ethnic groups were included.

### Spirometry

Lung function was assessed using a MasterScreen-PFT spirometer (Carefusion, Hoechberg, Germany), with Sentry Suite software (Version 2.17). All spirometries were performed by one single trained, very experienced respiratory lab technician.

Spirometry was performed in a sitting position. A nose clip was used to prevent air leaks. Testing for a participant was performed until: 1) the participant was able to achieve a reproducible spirometry result; 2) a maximum of eight attempts had been obtained; or 3) the participant could not continue. Each manoeuvre was automatically assessed by computer, based upon acceptability and reproducibility criteria according to the American Thoracic Society (ATS) – European Respiratory Society (ERS) standards [9]. These criteria were used as a reference aid to ensure a good quality of spirometry during testing.

Reference values were applied according to GLI 2012 adjusting for the following ethnic origins: Caucasian, African, north-east Asian, south-east Asian and other [7]. If FEV1/FVC or FVC was found to be below the LLN, spirometry was repeated 10–15 min after administration of 4 × 100 μg of salbutamol via a metered-dose inhaler and a spacer.

Normal spirometry was defined by baseline FEV1/FVC ratio and FVC above LLN, representing the lower 5th percentile based on age, gender, height and ethnicity [10]. Chronic airway obstruction was defined as FEV1/FVC below LLN after bronchodilation (BD). Reversible airway obstruction was defined as FEV1/FVC below LLN before BD and above LLN after BD. In case FVC was below LLN before BD and normalised after BD, we suspected air trapping and also classified these subjects as reversible airway obstruction. Possible lung restriction was defined as FVC below LLN after BD [10]. Finally, the prevalence of airway obstruction was also assessed when defined by an FEV1/FVC ratio < 0.70.

### Respiratory risk factors, symptoms and history of respiratory diagnoses

Information on respiratory status was obtained by a face-to-face structured interview by the respiratory technician on the day of spirometry.

Smoking was categorised as current, former or never. Number of pack-years was defined as the number of cigarettes smoked per day divided by 20 multiplied by the number of years that the participant smoked. Exposure to second-hand tobacco smoke in childhood and in adulthood, as well as exposure to other fumes or smokes, was also assessed.

Respiratory symptoms such as cough, sputum production and shortness of breath according to the Modified Medical Research Council (mMRC) dyspnoea scale [11] were documented.

Self-reported history of the following was also noted: a medical diagnosis of asthma; chronic obstructive pulmonary disease (COPD), emphysema or chronic bronchitis; other respiratory diseases; or previous lung operations.

### Other covariates

Body weight and height were measured with participants standing without shoes in light indoor clothing. Weight was measured in kilograms to the nearest 0.1 kg using a Seca™ scale (Seca, Hamburg, Germany). Height was measured to the nearest 5 mm using a Seca™ height gauge (Seca, Hamburg, Germany). Body mass index (BMI) was defined as weight/height^2^ in kg/m^2^.

### Inclusion criteria for spirometry

Manoeuvres were judged by the lab technician and one of two authors (JF, AL) from the flow-volume curve display. Spirometries were included in the analysis if the following conditions were fulfilled: no artefacts, no abrupt termination, no glottis closure or cough, no leaks, no large back extrapolated volume, as well as a maximal continuous effort [12].

### Statistical methods

Statistical analysis was performed using Stata™ version 15.0 software (StataCorp, College Station, TX, USA). Participants’ characteristics were expressed as number (percentage) for categorical variables, and as average ± standard deviation or median [interquartile range] for continuous variables. Between-group comparisons were performed using chi-square or Fisher’s exact test for categorical variables, and student’s t-test, analysis of variance or Kruskal-Wallis nonparametric test for continuous variables. For categories of spirometry, post-hoc between-group comparisons of continuous variables were performed using Scheffe’s method; normal spirometry was considered as the reference. Multivariable analysis was conducted using multinomial (polytomous) logistic regression, with spirometry categories as the dependent variable and all covariates significantly associated in the bivariate analysis as independent variables. Normal spirometry was considered as the reference, and results were expressed as relative risk ratio and (95% confidence interval). Statistical significance was considered for a two-sided test with *p* < 0.05.

## Results

### Participant selection and clinical characteristics

**Fig. 1:**
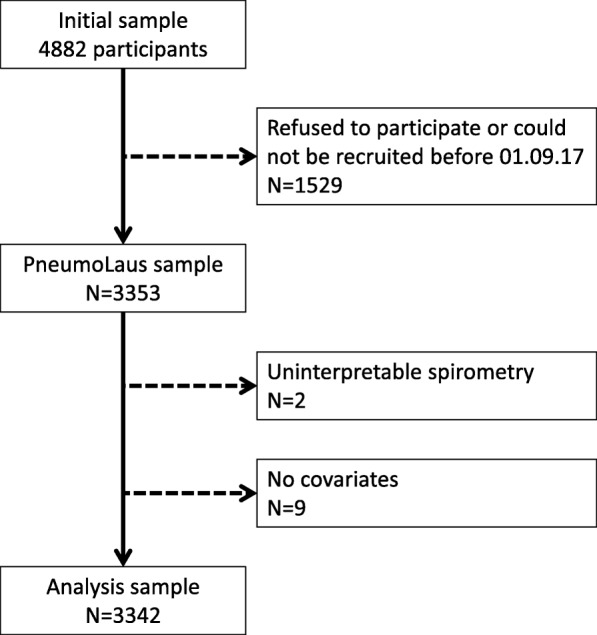
Selection procedure for the participants of the PneumoLaus study, 2014-2017, Lausanne, Switzerland

Of the initial 4882 eligible subjects, 3353 (68.7%) agreed to take part in the PneumoLaus study, and 3342 (68.5%) were included in the analysis. The selection procedure is summarized in Fig. 1. Baseline characteristics of the included participants as well as their gender distribution are shown in Table 1. There was a slight female preponderance; the vast majority of participants were Caucasian; mean BMI was ~ 26 kg/m^2^.

**Table 1:** Baseline characteristics of the participants of the PneumoLaus study, 2014–2017, Lausanne, Switzerland

Characteristics	Overall	Women	Men	*p*-value
Sample size	3342	1863	1479	
Age (years)	62.6 ± 10.0	63.2 ± 9.9	62.0 ± 10.0	< 0.001
Caucasian (%)	3261 (97.6)	1822 (97.8)	1439 (97.3)	0.347
Body mass index (kg/m^2^)	26.4 ± 4.7	25.8 ± 5.0	27.0 ± 4.0	< 0.001

Results are expressed as number of participants and (column percentage) for categorical variables and as average ± standard deviation for continuous variables. Between-group analysis performed using chi-square test for categorical variables and student’s t-test for continuous variables

### Overall spirometry results

In our sample, 91.9% participants had a normal spirometry. Airway obstruction was found in 6.3%: reversible in 2.5% and chronic in 3.8%. Possible lung restriction alone was present in 1.8%, and associated with chronic obstruction in 0.4%. There was only one case of reversible obstruction with possible restriction. Of all chronic airway obstructions, 58.6% could be classified as mild, 20.3% as moderate, 13.3% as moderately severe, 6.2% as severe and 1.6% as very severe [10]. When defined by FEV1/FVC < 0.70, airway obstruction was present in 12.3% of subjects. Among subjects with airway obstruction according to this fixed ratio but not according to the LLN (*n* = 224), 8.0% had a maximal mid-expiratory flow (MMEF25–75) below the LLN. This was higher than the prevalence of low MMEF25–75 in subjects with normal FEV1/FVC according to both definitions and lower than the prevalence of low MMEF25–75 in subjects with FEV1/FVC < LLN.

**Fig. 2:**
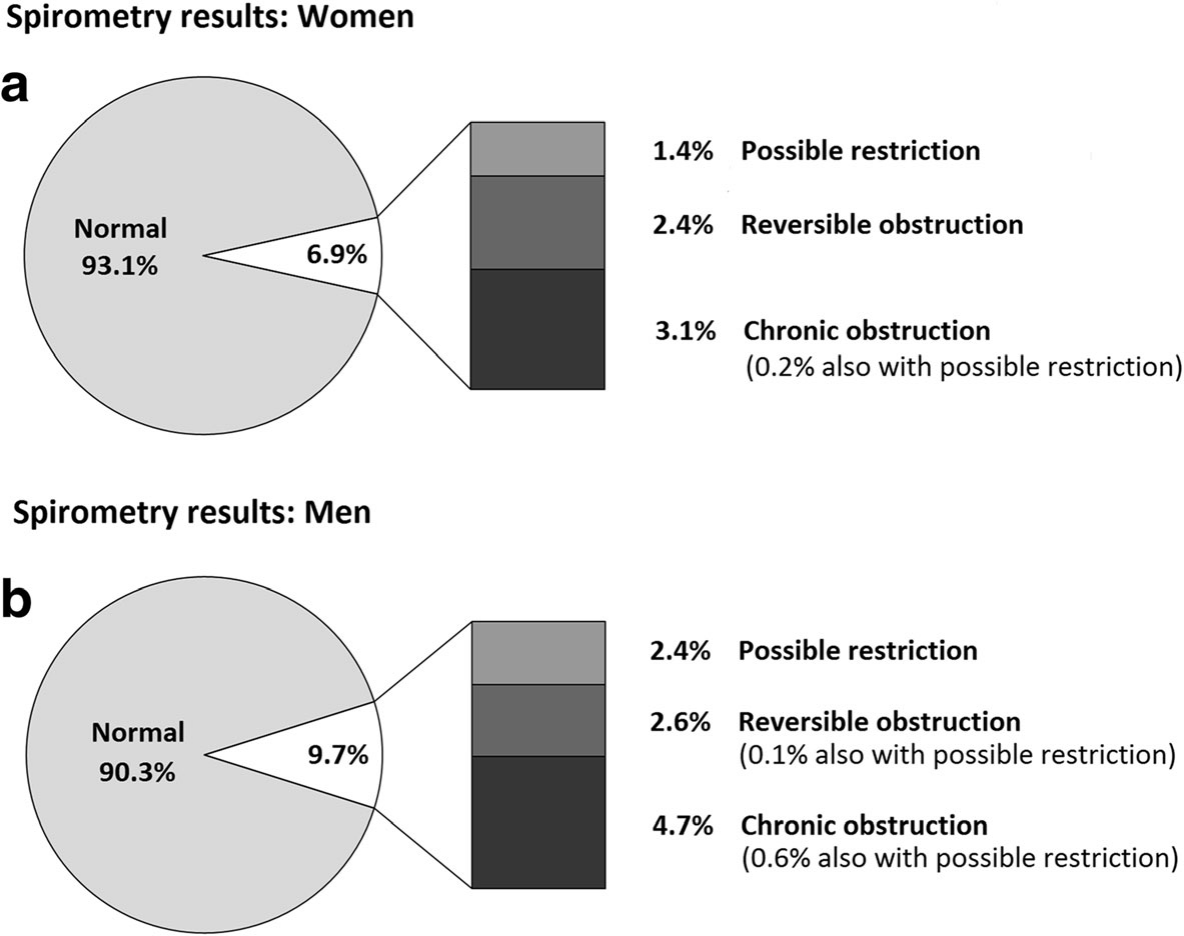
Spirometry results according to gender (M, male; F, female), Pneumolaus study, 2014-2017, Lausanne, Switzerland

Spirometry results by gender are shown in Fig. 2. Women were more likely to have normal spirometry (93.1% vs. 90.3%, *p* = 0.004), and had lower rates of possible lung restriction (1.6% vs. 3.0%, *p* = 0.005) and chronic obstruction (3.1% vs. 4.7%, *p* = 0.025). Relative risk ratio (RRR) for chronic obstruction in men compared to women was 1.59 (*p* = 0.02) according to multivariate analysis adjusting for all other variables; RRR for possible lung restriction in men was 1.80 compared to women (*p* = 0.037). Gender differences in spirometric categories persisted if applied to never-smokers only, with lower prevalence of possible lung restriction (1.7% vs. 4.0%, *p* = 0.003) and chronic obstruction (1.0% vs. 2.3%, *p* = 0.029) in female never-smokers. This difference disappeared when applied to ever-smokers only. There was no gender difference for reversible obstruction.

Average age did not differ significantly between spirometric result categories.

### Risk factors for respiratory diseases and their association with lung function abnormalities

Questionnaire results for respiratory risk factors according to spirometric categories are shown in Table 2. Former and current smokers constituted half of participants; they had a higher prevalence of abnormal spirometry (11.0% vs. 5.3%, *p* < 0.001), chronic obstruction (6.1% vs. 1.5%, *p* < 0.001) and reversible obstruction (3.5% vs. 1.4%, *p* < 0.001). There was no difference with regard to possible lung restriction in bivariate analysis (*p* = 0.183), and multivariate analysis suggested a protective effect of smoking for possible restriction. Overall, chronic airway obstruction was found in 8.9% of current smokers, 4.6% of former smokers and 1.5% of never smokers (*p* < 0.001). Among ever smokers, mean tobacco consumption was highest in the chronic obstruction category. Women were more often never-smokers (54.5% vs. 43.7%) and had less pack years if they were ever-smokers (21.5 vs. 27.5 pack years, *p* < 0.001).

**Table 2:** Respiratory risk factors according to spirometry results. PneumoLaus study, 2014–2017, Lausanne, Switzerland

Question	Overall	Normal spirometry	Chronic obstruction	Reversible obstruction	Possible restriction	*p*-value
N	3342	3070	128	83	61	
Smoking status:						< 0.001
Never	1662 (49.7)	1574 (51.3)	25 (19.5)	24 (28.9)	39 (63.9)	
Former	1075 (32.2)	975 (31.8)	49 (38.3)	37 (44.6)	14 (23.0)	
Current	605 (18.1)	521 (17.0)	54 (42.2)	22 (26.5)	8 (13.1)	
Ever smoker (%)	1680 (50.3)	1496 (48.7)	103 (80.5)	59 (71.1)	22 (36.1)	< 0.001
Mean pack years ^§ǂ^	20 [10–30]	19 [10–30]	33 [15–48]	20 [15–35]	25 [10–40]	< 0.001
Second-hand tobacco:						
Before age 18	1402 (42.0)	1295 (42.2)	54 (42.2)	26 (31.3)	27 (44.3)	0.253
As an adult	1707 (51.1)	1547 (50.4)	88 (68.8)	48 (57.8)	24 (39.3)	< 0.001
Other smoke / fumes	554 (16.6)	499 (16.3)	25 (19.5)	13 (15.7)	17 (27.9)	0.082
BMI (kg/m^2^)	26.4 ± 4.7	26.3 ± 4.6 ^a^	25.3 ± 4.6 ^a^	26.4 ± 4.4 ^a^	29.1 ± 6.8 ^b^	< 0.001

^§^ among ever smokers. *BMI* body mass index. Results are expressed as number of participants and (column percentage) for categorical variables and as average ± standard deviation or median [interquartile range] for continuous variables. Between-group analysis performed using chi-square test for categorical variables and analysis of variance or nonparametric Kruskal-Wallis test (ǂ) for continuous variables. Post-hoc between-group comparisons of BMI performed using Scheff’s method; values with differing superscripts are significantly different at *p* < 0.005. The group “chronic obstruction” also contains the subjects with both “chronic obstruction and possible restriction”. The group “reversible obstruction” also contains the subject with both “reversible obstruction and possible restriction”

Mean FEV1 as percentage of predicted was lower in ever-smokers compared to never-smokers (98.3% vs. 102.8%, *p* < 0.001) as were FVC (101.0% vs. 102.5%, *p* = 0.003) and the FEV1/FVC ratio (75.6% vs. 78.3%, *p* < 0.001).

Almost half of participants had been exposed to second-hand tobacco smoke in childhood, slightly more than half in adulthood, with no gender difference. In multivariate analysis, second-hand tobacco exposure before age 18 was associated with a protective effect for reversible obstruction (RRR = 0.50). Ever-smokers had more often experienced second-hand tobacco smoke exposure, both in childhood (47.3% vs. 36.6%, *p* < 0.001) and especially in adulthood (63.8% vs. 38.2%, *p* < 0.001).

With regard to other smokes and fumes, approximately 26% of men and 9% of women had been exposed (overall 16.6%), generally in relation to their professional environment (e.g. work as motor mechanics, professional cleaners, factory or kitchen workers). Subjects exposed to other smokes or fumes were more likely to have possible lung restriction compared with the rest of the population (3.1% vs. 1.6%, RRR = 1.87 in multivariate analysis), but were no different with regard to obstruction. Ever-smokers had more frequently been exposed to other smokes and fumes than never-smokers (19.3% vs. 13.8%, *p* < 0.001).

BMI was significantly higher in the group with possible restriction compared to the other groups.

Chronic airway obstruction was found in 1.5% of never-smokers, representing 19% of all chronic obstructions. These subjects did not differ from smokers with chronic obstruction with respect to mean age or gender distribution. They were more likely to have a history of asthma than smokers with chronic obstruction (36.0% vs. 16.5%, *p* = 0.049) but had no difference in prevalence of respiratory symptoms or other respiratory diagnoses.

### Respiratory symptoms and their association with lung function abnormalities

Questionnaire results for respiratory symptoms according to spirometric categories are shown in Table 3. Less than 5% of participants reported chronic respiratory symptoms: cough; sputum production; or dyspnoea rated mMRC ≥2. There was no gender difference in symptoms except that women more often reported slight dyspnoea on effort (mMRC =1) than men.

**Table 3:** Respiratory symptoms according to spirometry results. PneumoLaus study, 2014–2017, Lausanne, Switzerland

	Overall	Normal spirometry	Chronic obstruction	Reversible obstruction	Possible restriction	p-value
N	3342	3070	128	83	61	
Chronic cough	146 (4.4)	119 (3.9)	19 (14.8)	5 (6.0)	3 (4.9)	< 0.001
Chronic sputum production	82 (2.5)	58 (1.9)	17 (13.3)	4 (4.8)	3 (4.9)	< 0.001
Dyspnoea according to mMRC scale						< 0.001
0	1730 (51.8)	1635 (53.3)	45 (35.2)	33 (39.8)	17 (27.9)	
1	1563 (46.8)	1400 (45.6)	76 (59.4)	48 (57.8)	39 (63.9)	
≥ 2	49 (1.5)	35 (1.1)	7 (5.5)	2 (2.4)	5 (8.2)	

*COPD* chronic obstructive pulmonary disease, *mMRC* Modified Medical Research Council dyspnoea scale. Results are expressed as number of participants and (column percentage). Between-group analysis performed using Fisher’s exact test. The group “chronic obstruction” also contains the subjects with both “chronic obstruction and possible restriction”. The group “reversible obstruction” also contains the subject with both “reversible obstruction and possible restriction”

A considerable amount of subjects with chronic cough, chronic sputum production and chronic dyspnoea mMRC ≥2 had an abnormal spirometry result (18.5, 29.3 and 28.6% respectively). In particular, these participants showed more often chronic obstruction (14.3, 29.3 and 28.6% respectively). Conversely, compared to the rest of the population, subjects with chronic airway obstruction suffered more often from chronic cough (14.8% vs. 4.0%, *p* < 0.001), sputum production (13.3% vs. 2.0%, *p* < 0.001) and dyspnoea mMRC ≥2 (5.5% vs 1.3%, *p* < 0.001); but only sputum production stayed significantly increased after multivariate analysis (*p* = 0.005, RRR 3.91). In multivariate analysis, subjects with possible lung restriction were more often suffering from dyspnoea mMRC≥2 (*p* = 0.001, RRR 6.87).

The subjects defined as obstructive according to the fixed ratio but not obstructive according to LLN had a similar prevalence of chronic cough and sputum production to that of non obstructive subjects. Dyspnoea levels in these people were in between those of normal subjects and obstructive subjects according to LLN, but were not significantly different from either group (Table 4).

**Table 4:** Respiratory symptoms according to definition of obstruction. PneumoLaus study, 2014–2017, Lausanne, Switzerland

	Group 1: FEV1/FVC < LLN	Group 2: FEV1/FVC > LLN but < 0.7	Group 3: FEV1/FVC > LLN and > 0.7	p-value Group 1 vs. 2	p-value Group 2 vs. 3	*p*-value Group 1 vs. 3
N	188 (5.6)	224 (6.7)	2930 (87.7)			
Chronic cough	21 (11.2)	11 (4.9)	113 (3.9)	0.018	0.428	< 0.001
Chronic sputum production	18 (9.6)	7 (3.1)	56 (1.9)	0.006	0.208	< 0.001
Chronic dyspnoea mMRC ≥2	8 (4.3)	5 (2.2)	36 (1.2)	0.241	0.199	0.001

Results are expressed as number of participants and (percentage) for categorical variables. Between-group analysis performed using chi-square test. *mMRC* Modified Medical Research Council dyspnoea scale

Ever-smokers suffered more from chronic cough (5.7% vs. 3.1%, *p* < 0.001) and chronic sputum production (3.5% vs. 1.4%, *p* < 0.001) than never-smokers, even though overall prevalence of symptoms remained low. There was no difference with regard to dyspnoea mMRC ≥2 (1.8% vs. 1.1%, *p* = 0.122).

Higher BMI was associated with higher grade of dyspnoea: 30.9 kg/m^2^ in participants with dyspnoea mMRC ≥2 compared to 26.3 kg/m^2^ in participants with dyspnoea mMRC ≤1 (*p* < 0.001).

### History of respiratory diagnoses and their association with lung function abnormalities

Questionnaire results for self-reported respiratory diagnoses according to spirometric categories are shown in Table 5. Approximately 7% of women and 4% of men (overall 5.6%) indicated a medical diagnosis of asthma (*p* = 0.003); 25.7% of those had an abnormal spirometry result, 21.9% had an obstruction. There was no gender difference with regard to other known respiratory diseases.

**Table 5:** Self-reported respiratory diagnoses according to spirometry results. PneumoLaus study, 2014–2017, Lausanne, Switzerland

	Overall	Normal spirometry	Chronic obstruction	Reversible obstruction	Possible restriction	p-value
N	3342	3070	128	83	61	
Asthma	187 (5.6)	139 (4.5)	26 (20.3)	15 (18.1)	7 (11.5)	< 0.001
Chronic bronchitis, emphysema or COPD	53 (1.6)	29 (0.9)	22 (17.2)	2 (2.4)	0 (0)	< 0.001 ^a^
Any other pulmonary disease	40 (1.2)	27 (0.9)	4 (3.1)	2 (2.4)	7 (11.5)	< 0.001 ^a^
Previous lung operation	21 (0.6)	11 (0.4)	2 (1.6)	3 (3.6)	5 (8.2)	< 0.001 ^a^

*COPD* chronic obstructive pulmonary disease. Results are expressed as number of participants and (column percentage). Between-group analysis performed using chi-square test or Fisher’s exact test (^a^).The group “chronic obstruction” also contains the subjects with both “chronic obstruction and possible restriction”. The group “reversible obstruction” also contains the subject with both “reversible obstruction and possible restriction”

A medical diagnosis of chronic bronchitis, emphysema or COPD was reported by 1.6% of participants, and was more frequent amongst ever-smokers (2.7% vs. 0.5%, *p* < 0.001). Whilst more than half of them (54.7%) were normal according to their spirometry, a significant number of these “normal” individuals had chronic respiratory symptoms: 41.4% cough, 17.2% sputum production and 6.9% dyspnoea mMRC ≥2. There was no difference between smoking status categories with regards to history of asthma or other pulmonary diseases.

Chronic obstruction was associated with a medical diagnosis of asthma (RRR = 4.71) and chronic bronchitis, emphysema or COPD (RRR = 9.64) in multivariate analysis (Table 6). Similarly, reversible obstruction was associated with a diagnosis of asthma (RRR = 5.09) but also with previous lung operation (RRR = 9.57). Possible lung restriction was more prevalent in subjects with a diagnosis of asthma (RRR = 2.72), previous lung operation (RRR = 6.74) and any other pulmonary disease (RRR = 7.84).

**Table 6:** Multivariable analysis of the associations between clinical variables and spirometry results, Pneumolaus study, 2014-2017, Lausanne, Switzerland

	Chronic obstruction	p-value	Reversible obstruction	p-value	Possible restriction	p-value
Age (per year)	1.009 (0.989–1.028)	0.383	0.986 (0.964–1.009)	0.236	0.997 (0.971–1.024)	0.852
Gender (ref = woman)	1.589 (1.074–2.350)	0.02	1.187 (0.751–1.877)	0.463	1.796 (1.037–3.112)	0.037
Smoking status (ref = never)	3.495 (2.185–5.589)	< 0.001	2.734 (1.654–4.519)	< 0.001	0.534 (0.300–0.951)	0.033
Second-hand tobacco (ref = no)						
Before age 18	0.693 (0.467–1.028)	0.068	0.496 (0.305–0.806)	0.005	1.169 (0.679–2.014)	0.573
As an adult	1.496 (0.991–2.259)	0.055	1.142 (0.717–1.818)	0.575	0.618 (0.349–1.092)	0.097
Other smoke / fumes (ref = no)	0.799 (0.484–1.320)	0.382	0.750 (0.399–1.409)	0.371	1.874 (1.012–3.470)	0.046
Chronic cough (ref = no)	0.822 (0.333–2.025)	0.670	0.637 (0.175–2.324)	0.495	0.558 (0.100–3.104)	0.505
Chronic sputum production (ref = no)	3.907 (1.524–10.01)	0.005	2.392 (0.578–9.895)	0.229	1.846 (0.303–11.25)	0.506
Dyspnoea (ref = MRC < 2)	1.869 (0.665–5.250)	0.235	1.435 (0.319–6.467)	0.638	6.868 (2.309–20.43)	0.001
Self-reported diagnosis of (ref = no)						
Asthma	4.709 (2.745–8.077)	< 0.001	5.09 (2.760–9.386)	< 0.001	2.719 (1.172–6.308)	0.020
Chronic bronchitis, emphysema or COPD	9.637 (4.801–19.35)	< 0.001	1.876 (0.413–8.523)	0.415	NA	
Any other pulmonary disease	1.479 (0.385–5.688)	0.569	1.193 (0.207–6.872)	0.843	7.844 (2.597–23.70)	< 0.001
Previous lung operation (ref = no)	2.409 (0.425–13.67)	0.321	9.572 (2.046–44.78)	0.004	6.738 (1.682–26.99)	0.007

*NA* not assessable. Results are expressed as relative risk ratio and (95% confidence interval) using normal spirometry as the reference. The group “chronic obstruction” also contains the subjects with both “chronic obstruction and possible restriction”. The group “reversible obstruction” also contains the subject with both “reversible obstruction and possible restriction”

Only one third of subjects with chronic obstruction (32.8%) had a history of either: 1) asthma; or 2) chronic bronchitis, emphysema or COPD (6 participants had both). In fact, among all chronic airway obstructions, 64.1% of subjects were not aware of any respiratory disease at all. Whilst most of these people had no respiratory symptoms, 9.8% had chronic cough, 7.3% had chronic sputum production and 3.7% had dyspnoea mMRC ≥2.

## Discussion

The results of the PneumoLaus study reflect respiratory function in a sample of a Swiss city’s general population. Compared to similar studies, the prevalence of abnormal lung function was low in our study. Roughly speaking, we found 92% of subjects with normal spirometry, 6% with airway obstruction and 2% with possible lung restriction, with less than 1% showing a mixed ventilatory pattern. In the obstructive group, more than one third was reversible after bronchodilation, a proportion already observed in previous studies.

### Comparison with previous population studies

Similar population studies have been reported, such as the international Burden of Lung Disease (BOLD) Study [13], the European Respiratory Society Spirometry Tent [14], the Rotterdam Study in the Netherlands [15] or the Swiss study on Air Pollution and Lung Diseases In Adults (SAPALDIA) [16]. BOLD was a multicentre study, assessing the prevalence of airway obstruction using the NHANES III reference values [6], and employing bronchodilation with 200 μg salbutamol in all subjects. The prevalence of chronic airway obstruction was geographically heterogeneous, ranging from 5.7 to 23.0% for men and from 1.8 to 20.7% for women, but overall notably higher than the 3.9% found in our study. Similarly, the prevalence of possible lung restriction was higher in BOLD (8.4 to 67.7% in men, 8.4 to 68.7% in women) than in our study (2.2%). In the ERS spirometry tent study, spirometry was performed in volunteers in six European cities during ERS congresses from 2004 to 2009. No post-bronchodilation spirometry was performed. Overall prevalence of obstruction was 12.4% as defined according to LLN derived from population-specific prediction equations and 20.3% as defined by GOLD. In the Rotterdam study, obstruction was defined according to GOLD by a fixed ratio FEV1/FVC < 0.70, and no reversibility test was performed. The prevalence of obstruction was 4.7% at baseline and 13.6% in 2008 [17]. SAPALDIA is a multicentre study set in eight different Swiss regions, representing environmentally distinct conditions, investigating long-term effects of low-to-moderate air pollution. In the original study, bronchodilation was applied with 200 μg salbutamol if obstruction was found (FEV1/FVC < 80% predicted or FEV1 < 70%), which was the case of 4.7% of participants. In the first follow-up study (SAPALDIA 2), no bronchodilation was applied, and the investigators found 10.0% prevalence of obstruction [18], more than the 6.3% before bronchodilation in our study. Reference values for SAPALDIA 2 were Swiss [19].

In search of an explanation for our low rate of abnormal results, several factors have to be taken into account. In our population, half of participants were former or current smokers, less than in most other population studies. In the SAPALDIA study, 64.2% participants were former or current smokers at baseline [16], and this was the case of 63.4% participants in the Rotterdam Study [15]. The proportion of ever-smokers was also higher in Hannover (70.0%) and in Salzburg (59.4%), the closest BOLD sites to Lausanne, with prevalence rates for chronic obstruction of 18.1 and 26.6% respectively [20]. It might therefore be that the comparatively low rate of ever-smokers in PneumoLaus partly explains the lower prevalence of chronic obstruction. Nevertheless, the rate of ever-smokers in the ERS spirometry tent study was very close to ours (48.8%) but their rate of obstruction was still significantly higher.

### Impact of current reference values and guidelines

As expected in a population of this age range, the proportion of airway obstructions was lower using the LLN than the 0.70 fixed ratio for FEV1/FVC. Although the definition of airway obstruction by the LLN is rational, it has been argued that the 0.70 fixed ratio might detect subjects evolving towards obstruction but not yet fulfilling the LLN criteria [21]. For this intermediate group, obstructive according to the fixed ratio but not according to the LLN, our results show that these individuals behave like non-obstructive subjects with regard to chronic cough and chronic sputum production. Prevalence of dyspnoea mMRC ≥2 in this intermediate group falls between the normal and obstructive by LLN subjects with no significant difference from either of them. Since the difference between normal and obstructive by LLN subjects is significant (*p* = 0.001), this hints at a continuum between the three groups, although this should be interpreted with caution given the small size of these subgroups.

We followed the ATS/ERS recommendation for bronchodilation and used a dose of salbutamol twice as high as in the BOLD study, which may further contribute to a lower prevalence of chronic obstruction. Finally, in contrast to the above-mentioned studies, we used the Global Lung Function Initiative (GLI) 2012 reference values. Nevertheless, these are very similar to NHANES III reference values used by BOLD and to the Swiss reference values used by SAPALDIA. If anything, the use of GLI 2012 should result in a higher prevalence of obstruction due to a slightly higher LLN [7, 22]. The hypothesis that a particular characteristic of our population is responsible for these differences is supported by the fact that a recent study in Northern Italy also found a higher prevalence of chronic obstruction than ours (9.1%), despite a population sample with comparable smoking habits (ever-smokers 46%) and a very similar methodology to our own: use of GLI 2012 reference values and bronchodilation with 400 μg salbutamol if FEV1/FVC was lower than the LLN [23].

### Participant characteristics

We found more abnormal spirometry results in men than in women but this difference disappeared when analysis was applied to ever-smokers only, even though women are suspected to be at greater risk of smoking-induced lung function impairment [24]. This points to the potential role of other factors contributing to the development of chronic lung diseases [25]. Indeed, occupational exposure to dust, fumes and smoke was considerably higher in men in our population (26% vs. 9% in women). In terms of spirometry, subjects with occupational exposure more often had possible lung restriction.

BMI was higher in participants with possible lung restriction. This reflects similar results in European and Swiss cohorts [26] even though the exact relationship between obesity and the restrictive spirometric pattern is complex, and only a minority of obese subjects effectively display this pattern [27].

Only one third of our participants displaying chronic airway obstruction were aware of a respiratory disease. This observation highlights the well-known problem of COPD underdiagnosis [28, 29]. On the other hand, more than half of the subjects who reported a diagnosis of COPD, chronic bronchitis or emphysema had normal spirometry results, pointing either to COPD misdiagnosis, or to the possibility that diagnosis was mainly driven by respiratory symptoms [30].

### Strengths and limitations

The main limitation of our study consists of our population sample being part of the second follow-up of a cohort study. This implies a possible selection bias of particularly motivated and health-interested participants and might reflect in the relatively low proportion of ever-smokers [31, 32]. The cross-sectional design of the study limits the questions which can be addressed in this paper but should not strictly affect the quality of the data or the main results.

The main strengths of the PneumoLaus study are the use of the GLI 2012 reference equations, allowing to optimally adjust spirometry results for age and ethnicity as well as height and gender; the strict application of ATS/ERS criteria for bronchodilation, using a full salbutamol dose and therefore permitting to correctly classify obstruction into chronic and reversible; and the performance of all spirometries by one single laboratory technician, avoiding inter-observer variability.

## Conclusion

This study shows an unusually low rate of abnormal spirometry results in the general population of Lausanne, a medium-sized Swiss city, using GLI 2012 reference values. This is not explained by differences in design or methods when compared to similar population studies, but seems to be an inherent characteristic of our population.

## Abbreviations

ATS: American Thoracic Society
BD: Bronchodilation
BMI: Body Mass Index
BOLD: Burden of Lung Disease Study
COPD: Chronic Obstructive Pulmonary Disease
ECCS: European Community of Coal and Steel
ERS: European Respiratory Society
FEV1: Forced Expiratory Volume in 1 s
FVC: Forced Vital Capacity
GLI: Global Lung Function Initiative
GOLD: Global Initiative for Chronic Obstructive Lung Disease
LLN: Lower Limit of Normal
MMEF25–75: Maximal Mid-Expiratory Flow
mMRC: Modified Medical Research Council
NHANES: National Health and Nutrition Examination Survey
RRR: Relative Risk Ratio
SAPALDIA: Swiss study on Air Pollution and Lung Diseases In Adults
